# Surveillance system for coronavirus disease 2019 epidemiological parameters in Croatia

**DOI:** 10.3325/cmj.2020.61.481

**Published:** 2020-12

**Authors:** Krunoslav Capak, Robert Kopal, Tomislav Benjak, Ivan Cerovečki, Željka Draušnik, Lovro Bucić, Ivan Pristaš, Jelena Curać

**Affiliations:** 1 Croatian Institute of Public Health, Zagreb, Croatia; 2 University of Mostar School of Medicine, Mostar, Bosnia and Herzegovina; 3 Effectus University College, Zagreb, Croatia; 4 University of Rijeka School of Medicine, Rijeka, Croatia *tomislav.benjak@hzjz.hr*; 5 Croatian Health Insurance Fund, Zagreb, Croatia

The Croatian Institute of Public Health (CIPH), as the institution responsible for national health statistics, was in early March 2020 confronted with a serious challenge. After the official declaration of the coronavirus disease 2019 (COVID-19) epidemic, CIPH had to provide adequate resources for the surveillance of a wide array of epidemiological parameters on a short notice, which was necessary to manage the current public health crisis. It is noteworthy that at the time, Croatia, like many other countries in the European Union and worldwide, did not have a surveillance system established for this particular purpose. Fortunately, the Croatian health authorities managed to mobilize a broad inter-sector cooperation under CIPH coordination and established a functional surveillance system; this system now provides information to the National Civil Protection Directorate (NCPD), county Civil Protection Directorates, European institutions (such as the European Centre for Disease Prevention and Control), expert workgroups, national media, etc.

The basic data set produced on a daily basis for NCPD purposes encompasses the following: (i) the total number of SARS-CoV-2-infected patients in Croatia since the start of the epidemic, (ii) the number of newly diagnosed COVID-19 patients within the last 24 hours, including their distribution across Croatian counties, (iii) the total number of individuals tested for SARS-CoV-2, (iv) the number of individuals tested for SARS-CoV-2 within the last 24 hours, (v) the proportion of SARS-CoV-2-positive patients in the total number of individuals tested, and (vi) the distribution of SARS-CoV-2-infected patients in Croatia by sex, age groups, and counties of residence.

This data set is generated using the central SARS-CoV-2 database, established by the Croatian Health Insurance Fund in cooperation with CIPH. All of the 27 diagnostic laboratories that are running SARS-CoV-2 PCR testing submit the test results to the central test result database. The database collects data on both positive and negative results, as well as patients’ personal information. Data from the hospital care system are submitted to NCPD as well, encompassing the following: (i) the total number of patients undergoing hospital treatment for COVID-19, (ii) the number of patients admitted for hospital treatment for COVID-19 within the last 24 hours, (iii) the number of patients discharged from hospital treatment for COVID-19 within the last 24 hours, (iv) the total number of COVID-19 patients receiving mechanical ventilation support, (v) the number of COVID-19 patients put on mechanical ventilation support within the last 24 hours, (vi) the total number of fatalities due to COVID-19, and (vii) the number of fatalities due to COVID-19 within the last 24 hours. The data are collected using the LimeSurvey system, with access distributed by CIPH across 56 hospitals in Croatia. Data are delivered on an everyday basis and used to a form a daily report.

Croatian health authorities also maintain records on individuals in self-isolation. In order to monitor this and other relevant parameters (treatment at home, hospital treatment), based on the decision of the Croatian government, a digital platform was created. This database contains data on all health supervision procedures conducted on SARS-CoV-2-positive patients and their contacts by epidemiological teams, general practitioners, the Ministry of the Interior, and the State Inspectorate of the Republic of Croatia. The access to this platform is also granted to hospital physicians. The platform was designed to host records containing all the necessary parameters for every individual undergoing a health supervision procedure. All stakeholders using the platform are granted access to the data relevant to their access. The data contained in this platform are used to generate reports on the number of self-isolated individuals for NCPD, but also for the purposes of the Ministry of Justice and Public Administration, such as issuing of travel passes, which cannot be issued to individuals in self-isolation.

Beside the mentioned information, CIPH also produces data on the number of SARS-CoV-2-infected health care workers and the number of health care workers in self-isolation, as well as on their distribution by health facilities and professions. These data are generated by matching patient personal identification numbers contained in the central test result database with entries in the Croatian Healthcare Worker Registry. The presence of personal identification numbers in the central test result database has allowed for linking of this database with other registries administered by CIPH, eg, the Disabled Persons Registry, making it possible to monitor the epidemiological situation in this particularly vulnerable population group. Data from primary health care and hospital settings have been linked as well, giving researchers insight into pre-existing comorbidities of deceased patients and patients requiring mechanical ventilation support.

In collaboration with the Ministry of Science and Education, CIPH also monitors the data on the number of SARS-CoV-2-infected and self-isolated students and teachers. These data are obtained by matching personal identification numbers contained in the central test result database with those in the electronic register containing information on all students and teachers in elementary and high schools. The majority of these activities are performed by the CIPH Medical Informatics and Statistics Division, employing experts educated in data science.

The establishment of this surveillance system provided the basis for a timely and adequate epidemic response. The longer-term evaluation also allows for monitoring of various trends and projections development, which are useful for concurrent decision-making and preparations toward anticipated events.

